# Defining the threshold: triglyceride to high-density lipoprotein cholesterol (TG/HDL-C) ratio’s non-linear impact on tubular atrophy in primary membranous nephropathy

**DOI:** 10.3389/fendo.2024.1322646

**Published:** 2024-01-24

**Authors:** Mijie Guan, Liling Wu, Yuan Cheng, Dongli Qi, Jia Chen, Haiying Song, Haofei Hu, Qijun Wan

**Affiliations:** ^1^Department of Nephrology, Shenzhen Second People’s Hospital, Shenzhen, Guangdong, China; ^2^Department of Nephrology, The First Affiliated Hospital of Shenzhen University, Shenzhen, Guangdong, China

**Keywords:** triglyceride to high-density lipoprotein cholesterol ratio, tubular atrophy, primary membranous nephropathy, non-linear, cross-sectional study

## Abstract

**Background:**

Hyperlipidemia is common in primary membranous nephropathy (PMN) patients, and tubular atrophy (TA) is an unfavorable prognostic factor. However, the correlation between the triglyceride to high-density lipoprotein cholesterol (TG/HDL-C) ratio and TA is controversial. Therefore, our study aimed to investigate the association between the TG/HDL-C ratio and TA in PMN patients.

**Methods:**

We conducted a cross-sectional study and collected data from 363 PMN patients at Shenzhen Second People’s Hospital from January 2008 to April 2023. The primary objective was to evaluate the independent correlation between the TG/HDL-C ratio and TA using binary logistic regression model. We used a generalized additive model along with smooth curve fitting and multiple sensitivity analyses to explore the relationship between these variables. Additionally, subgroup analyses were conducted to delve deeper into the results.

**Results:**

Of the 363 PMN patients, 75 had TA (20.66%). The study population had a mean age of 46.598 ± 14.462 years, with 217 (59.78%) being male. After adjusting for sex, age, BMI, hypertension, history of diabetes, smoking, alcohol consumption, UPRO, eGFR, HB, FPG, and ALB, we found that the TG/HDL-C ratio was an independent risk factor for TA in PMN patients (OR=1.29, 95% CI: 1.04, 1.61, P=0.0213). A non-linear correlation was observed between the TG/HDL-C ratio and TA, with an inflection point at 4.25. The odds ratios (OR) on the left and right sides of this inflection point were 1.56 (95% CI: 1.17, 2.07) and 0.25 (95% CI: 0.04, 1.54), respectively. Sensitivity analysis confirmed these results. Subgroup analysis showed a consistent association between the TG/HDL-C ratio and TA, implying that factors such as gender, BMI, age, UPRO, ALB, hypertension and severe nephrotic syndrome had negligible effects on the link between the TG/HDL-C ratio and TA.

**Conclusion:**

Our study demonstrates a non-linear positive correlation between the TG/HDL-C ratio and the risk of TA in PMN patients, independent of other factors. Specifically, the association is more pronounced when the ratio falls below 4.25. Based on our findings, it would be advisable to decrease the TG/HDL-C ratio below the inflection point in PMN patients as part of treatment strategies.

## Background

Primary membranous nephropathy (PMN) is the leading pathological subtype of nephrotic syndrome (NS) in adults, characterized by proteinuria, hypoalbuminemia, edema, and hyperlipidemia (1). PMN is an immune-complex-mediated disorder, with 60-80% of patients exhibiting NS, where dyslipidemia is a defining feature, marked by high cholesterol and triglyceride (TG) levels (2). In PMN, significant proteinuria triggers hepatic overproduction of proteins and lipoproteins. The lipid and lipoprotein abnormalities in NS are mainly due to decreased clearance, as NS causes deficits in lipoprotein lipase, hepatic lipase, and the very-low-density lipoprotein (VLDL) receptor, while increasing cholesteryl ester transfer protein and the low-density lipoprotein (LDL) receptor-related protein. Additionally, alterations in lipoprotein structure hinder their receptor binding, activation of lipolytic enzymes, and exchange with high-density lipoprotein cholesterol (HDL-C) for lipid and apoprotein transfer (3). Hyperlipidemia results in lipid accumulation in kidney tubules, prompting an inflammatory response, oxidative stress, and subsequent cell damage, a process termed lipotoxicity. Tubular lipotoxicity contributes to a cascade of renal injuries, including oxidative stress, endoplasmic reticulum stress, tubular epithelial cell apoptosis, tubulointerstitial fibrosis, mitochondrial dysfunction, and inflammation (4). Tubular atrophy (TA) is a well-documented factor in the progression of renal disease (5).

In a rat model of diabetic nephropathy, there was a discovered link between TG accumulation in the kidneys and interstitial fibrosis in the tubulointerstitium (6). Additionally, pravastatin was shown to have a positive impact on reducing tubulointerstitial fibrosis in another rat model of cyclosporine-induced nephropathy (7). These findings emphasize the potential importance of lipid management in attenuating tubulointerstitial lesions in individuals with PMN.

TG levels, which are greatly influenced by feeding status, making it less reliable as a biomarker for prediction (8). The predictive value of high-density lipoprotein cholesterol (HDL-C) for cardiovascular disease (CVD) or mortality prediction remains a contentious issue, as evidenced by the ongoing debate in the medical literature (9). To address this uncertainty, researchers have suggested the use of the triglyceride to high-density lipoprotein cholesterol (TG/HDL-C) ratio as a more practical indicator for evaluating atherogenicity and insulin resistance (10, 11). The ratio has garnered attention for its enhanced predictive ability for CVD such as all-cause mortality, cardiac death, non-fatal myocardial infarction, stroke, or coronary revascularization procedures (10, 12, 13), as well as its importance in prognosing conditions like peritoneal dialysis and chronic kidney disease (CKD) (14).

According to a recent literature report, patients with IgA nephropathy who had a higher ratio of TG/HDL-C were observed to display more pronounced pathological lesions, such as interstitial fibrosis and TA (15). However, limited research has explored the potential link between the TG/HDL-C ratio and TA in patients diagnosed with PMN. Existing studies on dyslipidemia in PMN have mainly focused on its correlation with proteinuria, rather than its direct effect on renal outcomes (16).

Consequently, PMN presents a unique opportunity to investigate the TG/HDL-C ratio as a potential indicator of renal damage within a homogenous population where the primary disease process is well-defined, as opposed to a more heterogeneous group with varying causes of NS. To scrutinize this hypothesis, a retrospective cross-sectional study was carried out with 363 PMN participants, specifically examining the link between TG/HDL-C ratio levels and TA. Logistic regression modeling, smooth curve fitting, and a comprehensive suite of sensitivity analyses were employed to evaluate the relationship between the TG/HDL-C ratio and TA.

## Subjects and methods

### Study design

This cross-sectional study was conducted at a single center. In this particular research, the TG/HDL-C ratio was considered as the independent variable, while the dependent variable was TA (dichotomous variable: TA, non-TA).

### Study population

The researchers collected initial data for the study from kidney biopsy samples obtained during the hospitalization of qualified participants. Patients were screened based on clearly delineated inclusion and exclusion criteria to ensure their eligibility and suitability for the study sample. Obtaining biopsy data in this manner allowed the researchers to establish a baseline for key measures that would be tracked over the course of the study.

This cross-sectional study obtained data from individuals admitted to Shenzhen Second People’s Hospital from January 2008 to April 2023. The inclusion criteria consisted of patients diagnosed with membranous nephropathy via renal biopsy, and data collection followed a non-selective and consecutive approach. The researchers accessed the electronic case system and pathology reports to gather the essential information. It is crucial to highlight that the study strictly adhered to the principles outlined in the Declaration of Helsinki and obtained approval from the Medical Ethics Committee of Shenzhen Second People’s Hospital (ethical approval document number: 20210620213357018-FS01-02PJ).

**Figure 1:**
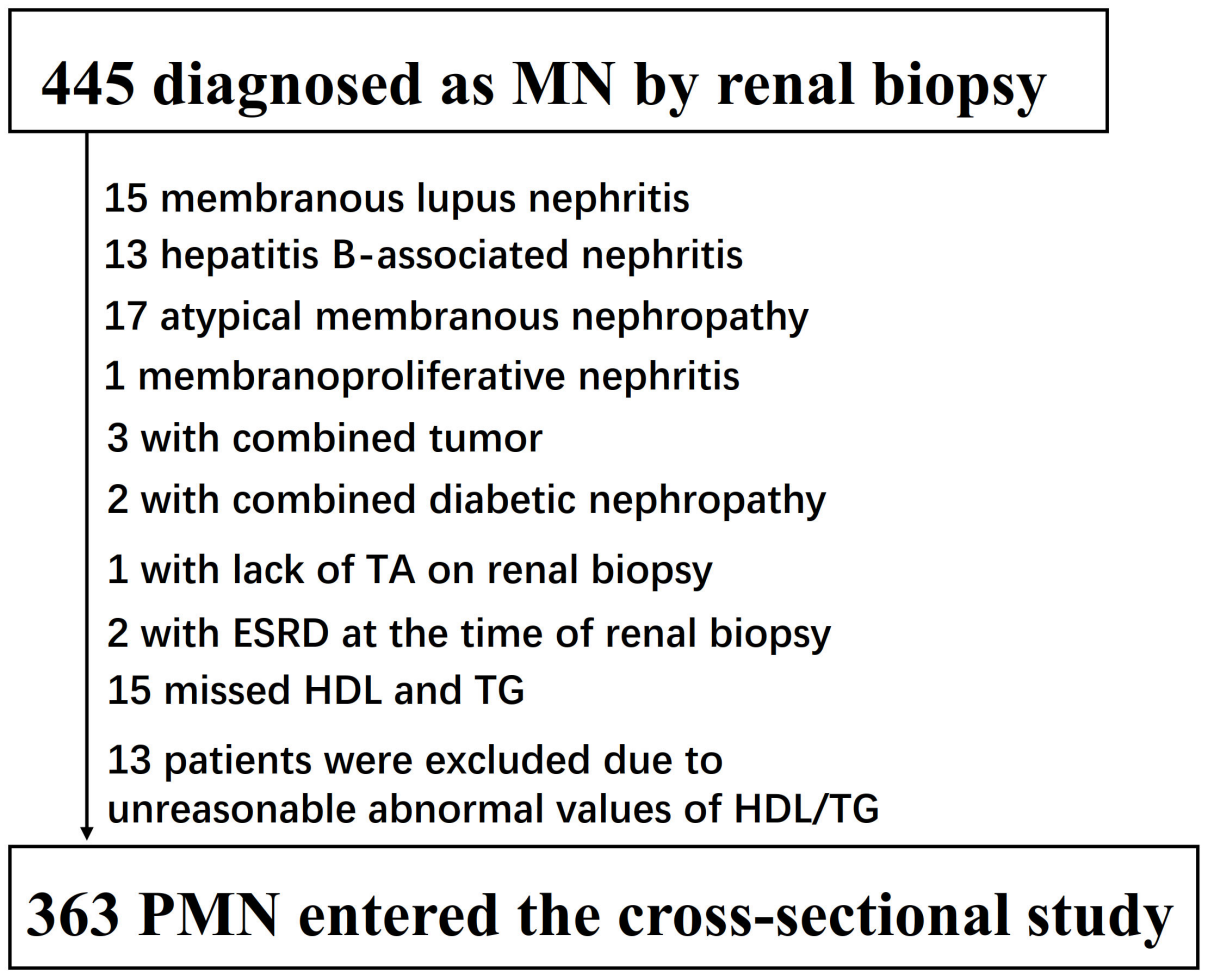
Flowchart of study participants. **Figure 1** showed the inclusion of participants. 445 participants were assessed for eligibility in the original study. We excluded patients with missing values of lupus nephritis (n=15), hepatitis B-associated nephritis (n=13), atypical membranous nephropathy (n=17), membranoproliferative nephritis (n=1), combined tumor (n=3), combined tumor (n=1), lack of TA on renal biopsy (n=1), missed HDL and TG (n=15), unreasonable abnormal values of TG/HDL-C (n=13), ESRD at the time of renal biopsy (n=2). The final analysis included 363subjects in the present study.

The initial enrollment of the cross-sectional study included 445 patients aged >14 years who were diagnosed with membranous nephropathy through renal biopsy. Patients were excluded if they met any of the following criteria: (1) renal pathology lacking TA description (1 patient); (2) cases of atypical membranous nephropathy, secondary membranous nephropathy, viral hepatitis, or malignancy (51 patients); (3) participants with missing data on HDL-c and TG levels (15 patients); (4) individuals with outliers in the TG/HDL-C ratio beyond the range of mean ± three standard deviations (13 patients); (5) patients with end-stage renal disease (ESRD) at the time of renal biopsy (2 patients). Consequently, 82 patients were excluded, leaving a final sample size of 363 patients with primary membranous nephropathy (PMN) who entered the cross-sectional study (see **Figure 1**). The patients were further divided into two groups based on the presence of TA.

Baseline data for the study were obtained from renal biopsies performed during hospitalization, ensuring that the patients satisfied the defined inclusion and exclusion criteria.

### Clinical data

During the initial renal biopsy, pertinent demographic data such as age, gender, blood pressure, hypertension, diabetes history, smoking and alcohol status, as well as body mass index, were carefully recorded. Moreover, essential laboratory measurements comprising serum creatinine, estimated glomerular filtration rate (eGFR), hemoglobin (HB), albumin (ALB), total cholesterol (TC), low-density lipoprotein cholesterol (LDL-C), HDL, triglycerides (TG), TG/HDL-C ratio, uric acid (UA), fasting plasma glucose (FPG) levels, 24-hour urine protein quantification (UPRO), and renal biopsy results were meticulously collected. The eGFR was evaluated by the CKD-EPI equation, providing valuable insights into renal function (17).

### Pathological data

The renal biopsy specimens underwent standard processing and examination techniques, including light microscopy, immunofluorescence, and electron microscopy. Pathological staging was conducted following the Ehrenreich-Churg system, which categorized stages into five distinct categories (18). Light microscopy utilized staining methods such as hematoxylin-eosin (HE), periodic acid-Schiff (PAS), hexosamine silver (PASM), and Masson staining. Various factors were assessed, including glomerular count, presence of spherical sclerosis, segmental sclerosis, crescent formation, mesangial proliferation, interstitial infiltration, and TA. Immunofluorescence was employed to observe deposits’ location and extent, such as IgG, IgA, IgM, C3, C1q, and PLA2R, using direct methods. Electron microscopy provided detailed insight into the ultrastructure, including the glomerular basement membrane, podocyte pedicles, and electron-dense material deposition. Renal pathology results were independently interpreted by two pathologists at the Guangzhou Jinyu Medical Laboratory Center.

### Variables

#### Triglyceride to high-density lipoprotein cholesterol ratio

The TG/HDL-C ratio was considered a continuous variable and calculated as follows: TG/HDL-C ratio = triglycerides divided by high-density lipoprotein cholesterol.

#### Tubular atrophy

In this study, the main focus was on the outcome variable TA, which was defined as a dichotomous variable categorized as 1 for TA and 0 for Non-TA. To assess TA, participants were diagnosed based on the criterion that the percentage of affected tubules ≥10% (19).

#### Covariates

Based on our clinical expertise and thorough review of relevant literature, we meticulously selected covariates for our study. These covariates, consisting of both continuous and categorical variables, have been previously identified in studies (5, 20) The continuous variables included age, BMI, 24-hour urine UPRO, eGFR, HB, FPG, and ALB. The categorical variables encompassed sex, hypertension, history of smoking, diabetes, and alcohol consumption. In order to ensure accuracy, we used the automated analyzer from Abbott AxSYM following standard protocols to assess the biochemical values. A physician conducted a comprehensive health habit inventory to gather pertinent information. BMI was calculated using weight in kilograms divided by the square of height in meters (kg/m^2^). We determined eGFR using the CKD-EPI equation (17) which takes into account age, gender, and creatinine levels. Data collection was carried out meticulously under standardized conditions, adhering to uniform procedures. Impaired kidney function was clinically defined as an estimated glomerular filtration rate (eGFR) < 60 ml/min/1.73m^2^ (21), while hypertension was determined by either documented history of hypertension or baseline systolic blood pressure ≥ 140 mmHg and/or diastolic blood pressure ≥90 mmHg.

### Statistical analysis

The participants were categorized based on the presence of TA. For continuous variables, we reported the mean ± standard deviation (SD) for those with a Gaussian distribution and the median (interquartile ranges) for those with a skewed distribution. As for categorical variables, we presented frequencies and percentages. We employed different statistical tests to compare the TA and non-TA groups. The χ2 test was used for categorical variables, independent samples t-test for variables with a normal distribution, and the Mann-Whitney test for variables with a skewed distribution. In our study, we encountered missing data points. Specifically, BMI data was missing for 33 participants (9.1%), TC data for 1 participant (0.27%), UPRO data for 3 participants (0.826%), history of alcohol data for 5 participants (1.38%), FBG data for 28 participants (7.71%), eGFR data for 1 participant (0.27%), and HB data for 11 participants (3.03%). To address these missing values, we performed multiple imputations (22). The imputation model included age, gender, DBP, SBP, BMI, hypertension, TC, UPRO, ALB, LDL-C, UA, FPG, eGFR, HB, history of alcohol, smoking, and diabetes. We conducted a missing data analysis assuming missing-at-random (MAR) conditions (23, 24).

### The linear association between TG/HDL-c ratio and TA

After conducting a collinearity screening process, we employed both univariate and multivariate binary logistic regression models in accordance with the guidelines outlined by the STROBE statement (24). These models enabled us to generate three unique variations: a non-adjusted model (referred to as the Crude model), a minimally-adjusted model (Model I), and a fully-adjusted model (Model II).

The Crude model did not involve any covariate adjustments. For Model I, we made adjustments solely for sociodemographic variables, including age, hypertension, sex, BMI, history of diabetes, smoking, and alcohol consumption. In Model II, we further adjusted for a comprehensive range of covariates presented in **Table 1**, such as age, hypertension, sex, BMI, history of diabetes, smoking, alcohol consumption, UPRO, eGFR, HB, FPG, and ALB. We recorded effect sizes (OR) alongside their corresponding 95% confidence intervals. If the introduction of covariates resulted in a change of 10% or more in the odds ratio (OR), we made necessary adjustments to ensure accuracy (24). These adjustments were informed by the outcomes derived from the collinearity screening process.

**Table 1:** Relationship between TG/HDL-c ratio and TA in different models.

Variable	Crude model (OR,95%CI, P)	Model I (OR,95%CI, *P*)	Model II (OR,95%CI, *P*)
**TG/HDL-c ratio**	1.34 (1.10, 1.64) 0.0034	1.30 (1.05, 1.61) 0.0156	1.29 (1.04, 1.61) 0.0213
TG/HDL-c ratio (Quintile)			
** Q1**	Ref.	Ref.	Ref.
** Q2**	2.27 (1.11, 4.67) 0.0255	1.92 (0.90, 4.12) 0.0921	1.71 (0.78, 3.76) 0.1800
** Q3**	3.52 (1.76, 7.05) 0.0004	3.20 (1.53, 6.70) 0.0021	2.99 (1.40, 6.36) 0.0046
**P for trend**	0.0003	0.0017	0.0035

Crude model: we did not adjust other covariants Model I: we adjusted age, hypertension, sex, BMI, history of diabetes, smoke, alcohol, Model II: we adjusted age, hypertension, sex, BMI, history of diabetes, smoke, alcohol, UPRO, eGFR, HB, FPG, ALB. OR, odds ratios; CI, confidence; Ref, reference.

### The non−linear association between TG/HDL-c ratio and TA

To address concerns about the adequacy of binary logistic regression models for handling nonlinear relationships, we utilized generalized additive models (GAM) and smooth curve fitting (penalized spline method) to examine the correlation between the TG/HDL-C ratio and TA. In case nonlinearity was detected, a recursive algorithm was applied to determine the inflection point. Subsequently, a two-piece binary logistic regression model was created for each side of the inflection point (25). A log-likelihood ratio test was then conducted to identify the most suitable model for describing the relationship between the TG/HDL-C ratio and TA. Furthermore, a generalized additive model (GAM) and smooth curve fitting were utilized to evaluate the non-linear relationship between the TG/HDL-C ratio and the incidence of TA in patients with normal kidney function. These methods enabled us to comprehensively explore and identify nuanced connections between these variables.

### Subgroup analysis

We conducted subgroup analyses using a stratified binary logistic regression model across various subgroups, including gender, hypertension, age, BMI, ALB, UPRO and severe nephrotic syndrome.

Firstly, we converted continuous variables such as age (<50, ≥50 years), BMI (<24, ≥24 kg/m^2^), ALB (<30, ≥30 g/L), UPRO (<3500, ≥3500 mg/24h) (26, 27), severe nephrotic syndrome ((edema, UPRO > 3500 mg/24h, ALB <25 g/L, hypertriglyceridemia) Yes, No) into categorical variables using established clinical thresholds.

Secondly, in addition to the stratification factor itself, we adjusted for all relevant factors, including age, hypertension, sex, BMI, history of diabetes, smoking, alcohol consumption, UPRO, eGFR, HB, FPG, and ALB. Finally, we conducted interaction tests using the likelihood ratio test to compare models with and without interaction terms (28, 29).

### Sensitivity analysis

To ensure the credibility of our findings, we undertook a comprehensive set of sensitivity analyses (30). Initially, we stratified the TG/HDL-c ratio into tertiles and performed trend tests to validate its use as a continuous variable while also investigating potential non-linear associations. Recognizing that individuals with impaired kidney function may face an elevated risk of TA (5), we conducted additional sensitivity analyses by exclusively including participants with normal kidney function to explore the linkage between the TG/HDL-c ratio and TA. Furthermore, we employed E-values (31) to assess the plausibility of unmeasured confounding factors influencing the relationship between the TG/HDL-c ratio and the risk of TA.

## Results

### Characteristics of patients

**Table 2** displays the demographic and clinical profiles of the participants included in this study. A total of 363 adults were analyzed, with 217 (59.78%) males, and an average age of 46.59 ± 14.46 years. The median TG/HDL-c ratio was 1.373 (0.907, 2.312), and the average BMI was 24.49 ± 4.0 kg/m^2^. The median UPRO was 3711.17 (2029.385, 6854) mg/24h and the mean eGFR was 103.23 ± 27.12 mL/min/1.73 m^2^. Among the participants, 75 patients (20.66%) were diagnosed with TA. Individuals with TA tended to be older, with higher systolic and diastolic blood pressure, TG levels, TG/HDL-C ratio, and lower eGFR. However, there were no significant differences between the two groups in relation to smoking status, diabetes history, alcohol consumption, gender, age, BMI, HB, FPG, ALB, uric acid, cholesterol, LDL, or HDL-C.

**Table 2:** The baseline characteristics of patients.

TA	Non- TA	TA	*P*-value
**N**	288 (52.06%)	75(47.93%)	
**GENDER**			0.582
**Male**	167 (57.99%)	50 (66.67%)	
**Female**	121 (42.01%)	25 (33.33%)	
**AGE(years)**	45.35 ± 14.60	51.39 ± 12.91	0.001
**BMI(kg/m^2^)**	24.36 ± 4.30	25.02 ± 2.66	0.207
**DBP(mmHg)**	81.12 ± 11.44	87.69 ± 15.14	<0.001
**SBP(mmHg)**	129.22 ± 18.84	137.56 ± 19.67	0.011
**ALB(g/L)**	27.47 ± 7.55	27.01 ± 6.75	0.634
**TC(mmol/L)**	7.10 ± 2.26	6.58 ± 2.05	0.069
**HDL.C(mmol/L)**	1.52 ± 0.75	1.52 ± 0.75	0.072
**TG(mmol/L)**	1.83 (1.33-2.65)	2.31 (1.66-3.25)	0.002
**LDL.c(mmol/L)**	4.72 ± 1.84	4.46 ± 1.54	0.261
**TG/HDL-c ratio**	1.28 (0.81-2.16)	1.88 (1.20-2.80)	<0.001
**UA(umol/L)**	385.06 ± 100.46	401.68 ± 90.44	0.194
**UPRO(mg/24h)**	3670.73 (2057.38-6557.84)	4201.66 (1873.34-7311.55)	0.596
**FPG(mmol/L)**	4.90 ± 0.91	5.02 ± 1.12	0.327
**eGFR (mL/min/1.73 m^2^)**	106.79 ± 25.83	89.53 ± 27.75	<0.001
**HB(g/L)**	132.30 ± 19.67	130.51 ± 23.06	0.499
**Smoke history**			0.582
**NO**	231 (80.21%)	58 (77.33%)	
**YES**	57 (19.79%)	17 (22.67%)	
**Alcohol history**			0.432
**NO**	243 (84.38%)	66 (88.00%)	
**YES**	45 (15.62%)	9 (12.00%)	
**Diabetes history**			0.459
**NO**	265 (92.01%)	67 (89.33%)	
**YES**	23 (7.99%)	9 (12.00%)	
**Hypertension**			<0.001
**NO**	162 (56.25%)	23 (30.67%)	
**YES**	126 (43.75%)	52 (69.33%)	

BMI, Body mass index; DBP, Diastolic blood pressure; SBP, Systolic blood pressure; ALB, albumin; TC, Total cholesterol; HDL-C, High-density lipoprotein cholesterol; TG, Triglyceride; LDL-C, Low-density lipid cholesterol l; TG/HDL-c ratio; Triglyceride to High-Density Lipoprotein Cholesterol ratio; UA, uric acid; UPRO, 24 h urine protein; FPG, Fasting plasma glucose; eGFR, evaluated glomerular filtration rate; HB, hemoglobin.

### Results of a binary logistic regression model used in univariate analyses

The univariate analyses revealed negative correlation between eGFR (OR = 0.980, 95% CI: 0.97-0.99), HDL-c 0.53 (OR = 0.980, 95% CI: 0.28-1.00) and TA, while positive correlations were observed with age (OR = 1.03, 95% CI: 1.01-1.05), DBP (OR = 1.04, 95% CI: 1.02-1.06), SBP (OR = 1.02, 95% CI: 1.01-1.04), TG/HDL-C ratio (OR = 1.34, 95% CI: 1.10-1.64), and TG (OR = 1.27, 95% CI: 1.06-1.52) (all P < 0.05; refer to **Table 3**). However, there was no significant associations between TA and gender, ALB, UA, UPRO, HB, FPG, TC, and LDL-C (all P > 0.05).

**Table 3:** The results of univariate analysis.

Variable	Statistics	OR (95%CI)	*P*-value
Gender			
**Male**	217 (59.780%)	Ref.	
**Female**	146 (40.220%)	0.69 (0.40, 1.18)	0.1734
**Age, years**	46.60 ± 14.46	1.03 (1.01, 1.05)	0.0015
**BMI(kg/m^2^)**	24.494 ± 4.020	1.04 (0.98, 1.11)	0.2075
**DBP(mmHg)**	82.482 ± 12.561	1.04 (1.02, 1.06)	0.0002
**SBP,mmHg**	130.939 ± 19.284	1.02 (1.01, 1.04)	0.0011
Smoke history			
**NO**	289 (79.61%)	Ref.	
**YES**	74 (20.386%)	1.19 (0.64, 2.19)	0.5823
Alcohol history			
**NO**	309 (85.12%)	Ref.	
**YES**	54 (14.876%)	0.74 (0.34, 1.58)	0.4334
Diabetes history			
**NO**	332 (91.46%)	Ref.	
**YES**	31 (8.540%)	1.38 (0.59, 3.21)	0.4609
Hypertension			
**NO**	185 (50.96%)	Ref.	
**YES**	178 (49.04%)	2.91 (1.69, 5.00)	0.0001
**ALB(g/L)**	27.373 ± 7.385	0.99 (0.96, 1.03)	0.633
**UA, umol/L**	388.50 ± 98.59	1.00 (1.00, 1.00)	0.194
**eGFR (mL/min/1.73 m^2^)**	103.226 ± 27.120	0.98 (0.97, 0.99)	<0.0001
**UPRO(mg/24h)**	5087.214 ± 4506.857	1.000 (1.000, 1.000)	0.3578
**HB, g/L**	131.932 ± 20.395	1.00 (0.98, 1.01)	0.4976
**FPG(mmol/L)**	4.923 ± 0.960	1.13 (0.88, 1.46)	0.3279
**TG/HDL-c ratio**	1.753 ± 1.200	1.34 (1.10, 1.64)	0.0034
**TC(mmol/L)**	6.993 ± 2.225	.89 (0.79, 1.01)	0.0695
**TG, mmol/L**	2.254 ± 1.287	1.27 (1.06, 1.52)	0.0112
**HDL-c(mmol/L)**	1.487 ± 0.696	0.53 (0.28, 1.00)	0.0486
**LDL-c(mmol/L)**	4.663 ± 1.782	0.92 (0.79, 1.06)	0.2604

Values are n (%) or mean ± SD or median (quartile) BMI, Body mass index; DBP, Diastolic blood pressure; SBP, Systolic blood pressure; ALB, albumin; UA, uric acid; eGFR, evaluated glomerular filtration rate; UPRO, 24 h urine protein; HB, hemoglobin; FPG, Fasting plasma glucose; TG/HDL-c ratio; Triglyceride to High-Density Lipoprotein Cholesterol ratio; TC, Total cholesterol; TG, Triglyceride; HDL-C, High-density lipoprotein cholesterol; LDL-C, Low-density lipid cholesterol.

### Analysis of multivariate data using binary logistic regression

The authors employed a binary logistic regression model to examine the correlation between the TG/HDL-C ratio and TA by constructing three distinct models. The findings indicated a significant association between the TG/HDL-C ratio and TA in the Crude model (OR=1.34, 95% CI: 1.1 - 1.64, P=0.0034). It was observed that each 1 unit increase in the TG/HDL-C ratio corresponded to a 34% increase in TA. In Model I, where only demographic variables such as age, hypertension, sex, BMI, history of diabetes, smoking, and alcohol consumption were adjusted for, each additional unit increase in the TG/HDL-C ratio was linked to a 30% elevated risk of TA (OR = 1.30, 95% CI: 1.05 to 1.61). Furthermore, in the fully adjusted Model II, which considered age, hypertension, sex, BMI, history of diabetes, smoking, alcohol consumption, UPRO, eGFR, HB, FPG, and ALB, each additional unit increase in the TG/HDL-C ratio was associated with a 29% increased risk of TA (OR = 1.29, 95% CI: 1.04 to 1.61). The confidence intervals of the results supported the reliability of the association between the TG/HDL-C ratio and the risk of TA as obtained from the model (**Table 1**).

### Sensitivity analysis

We performed a series of sensitivity analyses to validate the robustness of our findings. Firstly, we categorized the TG/HDL-C ratio into tertiles, transforming it from a continuous variable to a categorical one. This categorization was then reintroduced into the model. The results demonstrated that after this transformation, the effect sizes in different groups showed a consistent pattern, and the P-value for this trend remained in line with the results obtained when the TG/HDL-C ratio was treated as a continuous variable (**Tables 1**, **4**). Additionally, the authors calculated an E-value to evaluate the impact of unmeasured confounders on the results. The determined E-value was 1.53, surpassing both the relative risk associated with unmeasured confounders and the TG/HDL-C ratio. This suggests that the link between the TG/HDL-C ratio and the risk of TA remains largely unaffected by unknown or unmeasured confounding factors.

**Table 4:** Relationship between TG/HDL-c ratio and TA in patients with normal kidney function.

Variable	Crude model (OR,95%CI, *P*)	Model I (OR,95%CI, *P*)	Model II (OR,95%CI, *P*)
**TG/HDL-c ratio**	1.42 (1.16, 1.74) 0.0009	1.34 (1.08, 1.67) 0.0092	1.33 (1.06, 1.67) 0.0145
TG/HDL-c ratio (Quintile)			
** Q1**	Ref.	Ref.	Ref.
** Q2**	2.22 (0.98, 5.01) 0.0558	1.92 (0.82, 4.51) 0.1355	1.71 (0.72, 4.09) 0.2273
** Q3**	4.35 (2.03, 9.33) 0.0002	3.75 (1.67, 8.42) 0.0013	3.44 (1.50, 7.86) 0.0035
**P for trend**	<0.0001	0.0008	0.0022

**Table 4** was sensitivity analysis in participants with ml/min/1.73m^2^(n= 340). Crude model: we did not adjust other covariants Model I: we adjusted age, hypertension, sex, BMI, history of diabetes, smoke, alcohol, Model II: we adjusted age, hypertension, sex, BMI, history of diabetes, smoke, alcohol, UPRO, eGFR, HB, FPG, ALB. OR, odds ratios; CI, confidence; Ref, reference.

Furthermore, another sensitivity analysis was conducted where participants with impaired kidney function were excluded. Out of all the participants, 23 (6.3%) were identified as having impaired kidney function. The findings indicated that even after controlling for confounding factors, there still existed a positive association between the TG/HDL-C ratio and the risk of TA (OR = 1.33, 95% CI: 1.06 to 1.67) (**Table 4**).

### The nonlinearity addressed by the generalized additive model

**Figure 2:**
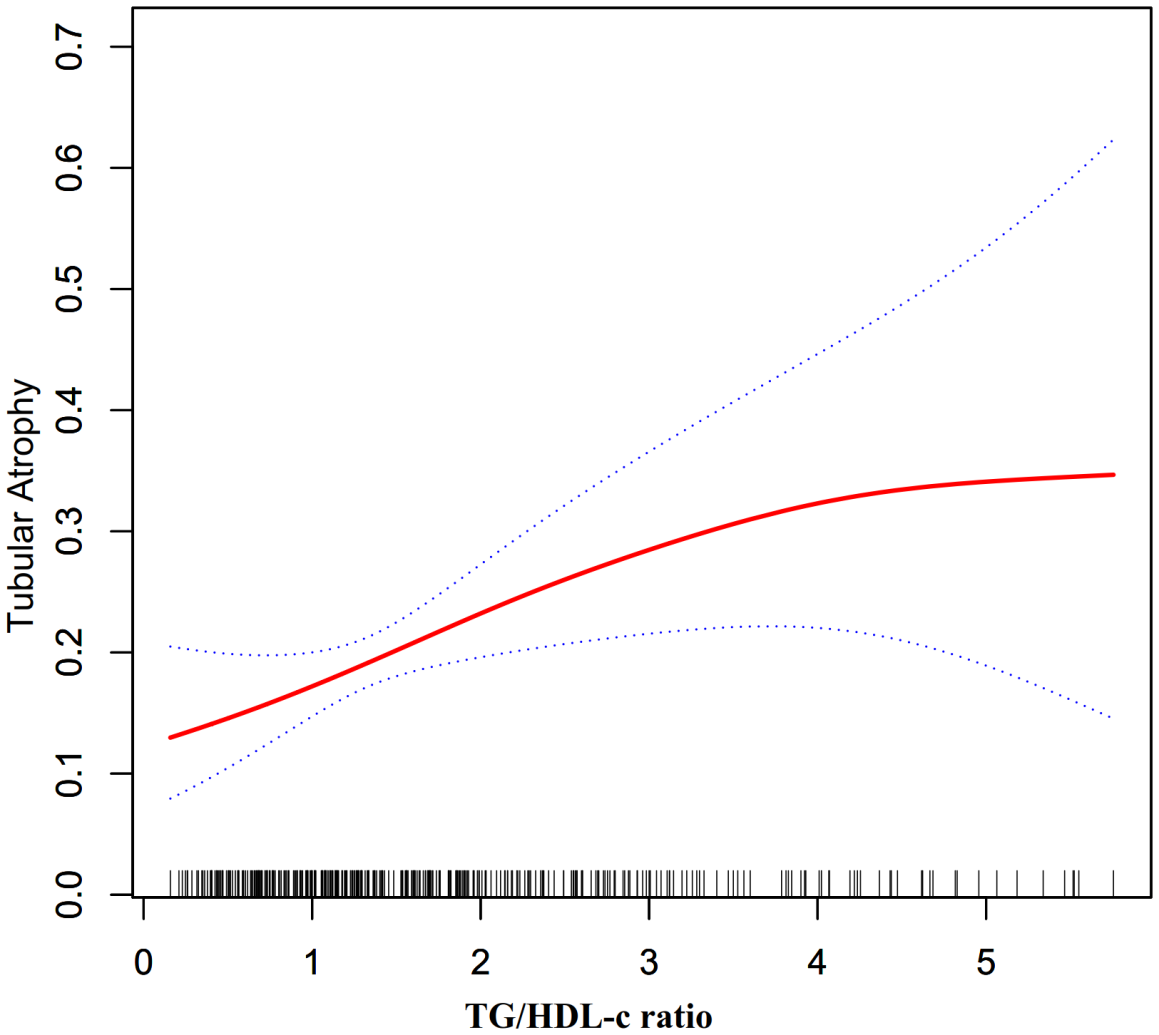
The non-linear relationship between TG/HDL-c ratio and the risk of TA.

Through the use of GAM and smooth curve fitting, we have discovered a nonlinear association between the TG/HDL-C ratio and TA (**Figure 2**). Employing a recursive algorithm, we accurately determined the inflection point to be at 4.25. Subsequently, our investigation applied a two-piece binary logistic regression model to calculate the effect size and establish the confidence intervals surrounding the inflection point. Notably, on the left side of the inflection point, the effect size and corresponding 95% CI values were 1.56 (1.17, 2.07), respectively. Conversely, on the right side of this pivotal point, the effect size and 95% CI values were 0.25 (0.04, 1.54), respectively (**Table 5**).

**Table 5:** The result of the two-piecewise Cox regression model.

Incident TA	Model I (OR,95%CI, *P*)	Model II (OR,95%CI, *P*)
**Fitting model by standard Cox regression**	1.29 (1.04, 1.61) 0.0213	1.33 (1.06, 1.67) 0.0145
Fitting model by two-piecewise Cox regression		
**Inflection point of TG/HDL-c ratio**	4.25	4.25
** ≤Inflection point**	1.56 (1.17, 2.07) 0.0023	1.65 (1.22, 2.23) 0.0013
** >Inflection point,**	0.25 (0.04, 1.54) 0.1356	0.23 (0.03, 1.47) 0.1193
**P for log-likelihood ratio test**	0.043	0.033

Model I: All participants; Model II: Participants with eGFR ≥ 60 ml/min/1.73m2; OR, odds ratios; CI, confidence; Ref, reference.

We adjusted age, hypertension, sex, BMI, history of diabetes, smoke, alcohol, Model II: we adjusted age, hypertension, sex, BMI, history of diabetes, smoke, alcohol, UPRO, eGFR, HB, FPG, ALB.

We adjusted age, SBP, sex, BMI, SBP, history of diabetes, hypertension, smoke and alcohol, UPRO, eGFR, HB, FPG, ALB. HR, Hazard ratios; CI, confidence; Ref, reference.

**Figure 3:**
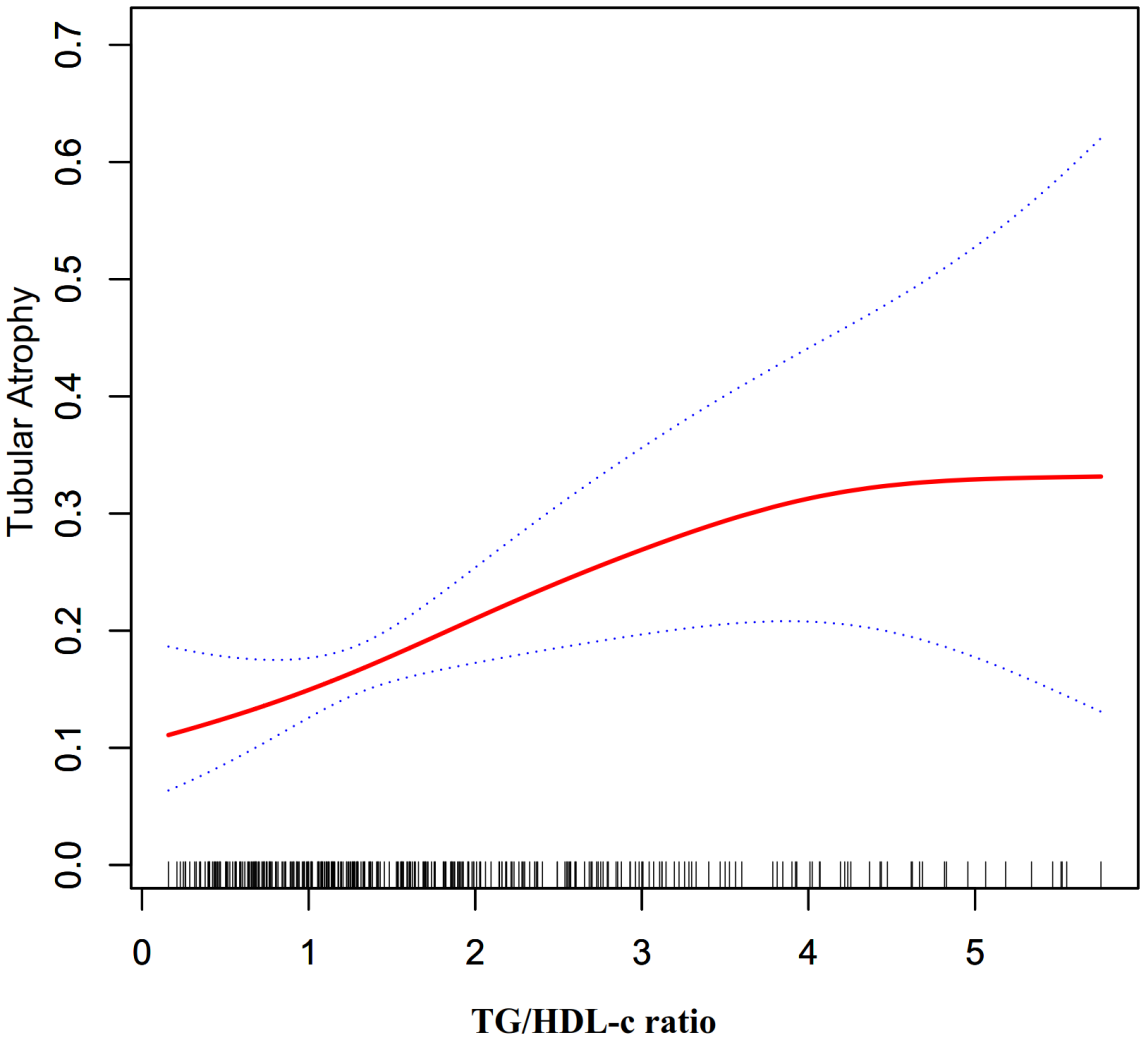
The non-linear relationship between TG/HDL-c ratio and the risk of TA in participants with normal kidney function.

Furthermore, an additional sensitivity analysis was exclusively conducted on participants with normal kidney function. After adjusting for various confounding variables including age, hypertension, sex, BMI, history of diabetes, smoking, and alcohol consumption, a comprehensive two-stage linear regression analysis revealed a non-linear relationship between the TG/HDL-C ratio and the risk of TA in PMN patients. The investigation effectively identified the inflection point for the TG/HDL-C ratio, precisely pinpointing it at 4.25. On the left side of this critical point, the odds ratio (OR) and its associated 95% CI were determined to be 1.65 (1.22, 2.23), respectively. On the opposite side, specifically the right side of the inflection point, the OR and corresponding 95% CI values were found to be 0.23 (0.03, 1.47), respectively (refer to **Table 5**; **Figure 3**).

### The results of subgroup analyses

The authors conducted a comprehensive subgroup analysis to explore the potential influence of various confounding factors, including hypertension, BMI, UPRO, and age, on the association between the TG/HDL-C ratio and TA risk. Stratification variables, namely gender, age, hypertension, ALB, BMI, and UPRO, and severe nephrotic syndrome were utilized to detect any identifiable patterns in effect sizes across these variables (**Table 6**). Interestingly, results from **Table 6** indicate that gender, age, hypertension, ALB, BMI, UPRO, and severe nephrotic syndrome do not significantly affect the relationship between the TG/HDL-C ratio and TA risk (All P for interaction >0.05). These findings suggest that the connection between the TG/HDL-C ratio and TA remains consistent and unchanged.

**Table 6:** Effect size of TG/HDL-c ratio on TA in prespecified and exploratory subgroups.

Characteristic	No of participants	HR (95%CI)	P-value	P for interacion
**Gender**				0.5155
** Male**	217	1.22 (0.92, 1.62)	0.1664	
** Female**	146	1.43 (0.96, 2.14)	0.0762	
**BMI (kg/m^2^)**				0.6062
** ≥24**	1462	0.970 (0.932, 1.009)	0.1292	
** <24**				
**Age (years)**				0.5868
** ≥50**	153	1.33 (0.97, 1.82)	0.0769	
** <50**	210	1.17 (0.85, 1.62)	0.3319	
**UPRO (mg/24h)**				0.7299
** ≥3500**	195	1.22 (0.90, 1.65)	0.2049	
** <3500**	168	1.32 (0.95, 1.84)	0.1019	
**ALB (g/L)**				0.9679
** ≥30**	132	1.31 (0.91, 1.89)	0.1451	
** <30**	231	1.33 (1.00, 1.75)	0.0473	
**Hypertension**				0.4625
** NO**	185	1.16 (0.78, 1.73)	0.4678	
** YES**	178	1.39 (1.05, 1.85)	0.0208	
**Heavy nephrosis**				0.4774
** NO**	288	1.436 (1.107, 1.861)	0.0063	
** YES**	75	1.107 (0.569, 2.155)	0.7653	

Note 1: Above model adjusted for age, hypertension, sex, BMI, history of diabetes, smoke, alcohol, UPRO, eGFR, HB, FPG, ALB.

Note 2: In each case, the model is not adjusted for the stratification variable

HR, Hazard ratios; CI, confidence; Ref, reference.

## Discussion

The objective of this cross-sectional study was to investigate the correlation between the TG/HDL-C ratio and TA among PMN patients. The findings indicated an independent association between the TG/HDL-C ratio and the risk of TA in this specific patient population. Notably, a threshold effect curve was observed, suggesting varying associations between the TG/HDL-C ratio and TA on either side of the inflection point among PMN patients, which was determined to be 4.25. Additional support for the robustness and consistency of these results was provided by sensitivity and subgroup analyses. Overall, these findings suggest that the TG/HDL-C ratio may serve as a valuable reference for the primary prevention of TA in PMN patients.

Among the 363 PMN patients included in this study, 75 (20.66%) were diagnosed with TA. These findings deviated from previous studies which reported a proportion of renal TA ranging from 52.6% to 66% in patients with PMN (5, 32, 33). The observed disparities may be attributed to several possible reasons. Firstly, differences in racial backgrounds among the study populations could have played a role. Secondly, inconsistencies in the criteria used to define tubular atrophy may have contributed to the variations in results. Importantly, it is noteworthy that the prevalence of TA was found to be high. Therefore, it remains crucial to actively explore additional risk factors associated with the development of TA. Our analysis revealed that patients with TA tended to be older, with hypertension, elevated TG levels, higher TG/HDL-C ratio, and lower eGFR.

After conducting a literature search, there was rare literature investigating the relationship between the TG/HDL-C ratio and TA risk. However, a recent retrospective cohort study at the West China Hospital of Sichuan University investigated this topic specifically in patients diagnosed with IgA nephropathy. The study included 1146 subjects with IgA nephropathy, divided into two groups based on their TG/HDL-C ratio at the time of renal biopsy: a high TG/HDL group (TG/HDL ≥ 1.495, N=382) and a low TG/HDL group (TG/HDL-C < 1.495, N=764). The study’s findings indicated that individuals with a higher TG/HDL-C ratio exhibited more severe pathological lesions with tubular atrophy/interstitial fibrosis (odds ratio [OR] 1.610, 95% confidence interval [CI] (1.203-2.154, P=0.001) (15).

In our recent cross-sectional study, we observed a higher TG/HDL ratio among individuals with TA. To investigate the potential association between TG/HDL-C ratio and TA risk, we conducted logistic regression analysis, carefully adjusting for numerous factors, including age, hypertension, sex, BMI, history of diabetes, smoking, alcohol consumption, UPRO, eGFR, HB, FPG, and ALB. The results revealed a significant positive relationship between TG/HDL-C ratio and TA risk (OR=1.29, 95% CI: 1.04, 1.61, P=0.0213). These findings are consistent with the earlier mentioned study and contribute to the existing literature by demonstrating that an elevated TG/HDL-C ratio increases the risk of TA, regardless of participants’ eGFR levels.

Importantly, our study stands out from previous research in several key aspects. Firstly, we meticulously adjusted for multiple confounding variables, a factor that the previous study overlooked as it solely relied on univariate logistic regression models. Secondly, our study specifically examined patients with a distinct kidney condition, namely individuals with PMN, thereby advancing the understanding of the relationship between the TG/HDL-C ratio and TA across different pathological types of kidney diseases. Moreover, through subgroup and sensitivity analyses, we found that this relationship remained consistent even among participants with normal kidney function. These comprehensive efforts have further strengthened the stability and reliability of the association between the TG/HDL-C ratio and TA risk. In summary, our study offers valuable insights into the correlation between the TG/HDL-C ratio and TA risk. The findings highlight the significance of clinical intervention targeted at managing TG/HDL-C ratio levels to mitigate the risk of TA in patients with PMN.

Additionally, in comparison to existing medical literature, our cross-sectional study has provided novel insights into the relationship between the TG/HDL-C ratio and TA risk. Notably, this study is the first to observe a non-linear association between these variables. To examine this complex relationship, our research team utilized a two-piecewise logistic regression model. Through meticulous adjustment for confounding factors, we determined the inflection point to be at 4.25. Intriguingly, our findings indicated that when the TG/HDL-C ratio was below 4.25, a 1-unit increase in the TG/HDL-C ratio level was accompanied by a significant 56% increase in the adjusted odds ratio (OR) of TA risk (OR = 1.56, 95%CI: 1.17–2.07). Conversely, when the TG/HDL-C ratio exceeded 4.25, no statistically significant correlation between the TG/HDL-C ratio and TA was observed.

The identification of a 4.25 TG/HDL-C ratio inflection point is indeed a novel finding. Lipid metabolism dysfunction may substantially impact renal health. In PMN patients specifically, intrinsic disease processes disrupt lipid homeostasis, potentially increasing TG and decreasing HDL-C plasma levels, thereby affecting the TG/HDL-C ratio. The non-linear relationship between the TG/HDL-C ratio and the risk of TA in PMN patients can be elucidated by several mechanisms. (1). Lipid toxicity (4, 34): At a certain threshold, the TG/HDL-C ratio may reflect a critical level of dyslipidemia that leads to lipid toxicity, this can result in the accumulation of free fatty acids and their toxic metabolites, causing oxidative stress and inflammation, which ultimately leads to tubular epithelial cell injury and tubulointerstitial damage. (2). Glomerular filtration of lipids (35): An increased TG/HDL-C ratio may cause lipids to be filtered by the glomeruli and subsequently taken up by tubular cells, inducing cellular stress, apoptosis, and contributing to tubulointerstitial changes. (3). Threshold effect: Our investigation reveals a TG/HDL-C ratio threshold 4.25, below this ratio, TA susceptibility rises sharply, while above, risk plateaus. This non-linear pattern likely stems from multiple factors. Foremost, rigorous statistical analysis determined the inflection point, controlling confounders that could impact the TG/HDL-C ratio and TA risk. Such findings imply that beyond a certain lipid-induced renal impairment level, ancillary factors may come into play in the injury’s progression. We found individuals with ratios ≤4.25 were predominantly female, without hypertension, and had lower BMIs, DBPs, UAs and UPROs compared to those with higher ratios (**Supplementary Table S1**). Although these features strongly associate with TA risk (5, 33, 36–38), their collective impact enhances among those with ratios >4.25, reducing the TG/HDL-C ratio’s effect. In contrast, with fewer impactful risk factors present below 4.25, the ratio’s influence is amplified—likely contributing to the nonlinear relationship. Recent studies have shown a nonlinear correlation between TG/HDL-C ratios and the risk of CKD in American adults, pinpointing the curve’s inflection point at a TG/HDL-C ratio of 6.68 (39). Furthermore, our literature review indicates that this ratio also exhibits a nonlinear relationship with the risk of other conditions, such as in-hospital mortality from acute type B aortic dissection, arterial stiffness, the incidence of type 2 diabetes, and the onset of pre-diabetes (40–43). These studies consistently reveal that as TG/HDL-C ratios increase, the risk of disease rises but then plateaus after exceeding a certain threshold, suggesting a saturation effect. This implies that beyond a certain point, further increases in the TG/HDL-C ratio do not proportionally escalate the risk of disease. The exact factors and mechanisms underlying this pattern warrant further exploration. Another possible explanation is that high TG/HDL-C ratios often lead clinicians to initiate lipid-lowering therapy, which could mitigate TG/HDL-C related damage to renal tubules through medical intervention (7).

Therefore, a non-linear relationship means changes in one variable do not consistently correspond to changes in another-their interrelation is complex, virtually absent and unpredictable, unlike linear relationships. However, non-linear entities can be related to each other in ways that are fairly predictable, but simply more complex than in a linear relationship. Given the intricate association between the TG/HDL-C ratio and TA risk, identifying this non-linear relationship brings us closer to elucidating their true connection. Pinpointing a TG/HDL-C threshold ratio of 4.25 holds major clinical implications: (1). Risk Stratification: The TG/HDL-C ratio serves to assess TA risk in PMN patients. Recognizing 4.25 as a potential inflection point for renal damage assists clinician risk appraisal and therapeutic timing. (2). Therapeutic Intervention: This non-linear relationship finding enables tailored lipid-lowering treatments. For example, statins/fibrates could be used more aggressively in patients exceeding this threshold to mitigate TA risk. (3). Monitoring and Management: Consistent tracking of the TG/HDL-C ratio in PMN patients can reveal the success of treatments and signal when adjustments in dyslipidemia management are necessary.

After conducting a thorough subgroup analysis, the authors found that age, BMI, hypertension, UPRO, ALB, and severe nephrotic syndrome do not function as effect modifiers in influencing the association between the TG/HDL-C ratio and TA. Irrespective of factors such as gender, age, BMI, blood pressure, albumin level, the presence of massive proteinuria or severe nephrotic syndrome, effectively controlling the TG/HDL-C ratio demonstrated a significant decrease in the risk of TA.

The potential mechanisms underlying renal damage caused by the TG/HDL-C ratio can be explained as follows. Firstly, the reabsorption of phospholipids and cholesterol in renal tubular epithelial cells leads to the release of inflammatory factors and tissue damage (44–46). Secondly, the accumulation of lipoproteins in the glomerular mesangium stimulates the production of inflammatory cytokines, activating macrophages and ultimately resulting in glomerulosclerosis (44). Thirdly, a high TG/HDL-C ratio is a risk factor for both atherosclerosis (47) and CKD (48). Additionally, the TG/HDL-C ratio proves to be a reliable indicator of insulin resistance (49), which in turn induces oxidative stress (50). This oxidative stress impairs the activation of nuclear factor erythroid-2-related factor-2, which is a protective mechanism against kidney tissue injury (51).

### Study strengths and limitations

Our study possesses several strengths. Firstly, we employed both categorical and continuous TG/HDL-C ratios as independent variables to assess their correlation with TA risk. This approach effectively minimizes information loss and allows for the quantification of their relationship. Secondly, we tackled missing data by utilizing multiple imputations, a method that enhances the statistical power and mitigates potential bias arising from missing covariate information. Thirdly, our study represents a significant advancement in the understanding of nonlinearity compared to prior research. Moreover, we have revealed a non-linear relationship between the TG/HDL-C ratio and the risk of TA in patients with PMN, thereby enhancing our understanding in this domain.

However, it is important to consider several limitations in our study. Firstly, our study focused exclusively on PMN patients, and therefore further validation is necessary to generalize these findings to other types of glomerulonephritis. In future studies, efforts should be made to validate the correlation between the TG/HDL-C ratio and TA in different types of glomerulonephritis. Secondly, we only examined the TG/HDL-C ratio at baseline, without considering any subsequent changes over time. Additionally, certain indicators related to TA, such as medication history, were not included in the original data. To address these limitations, we can adjust our study design or collaborate with other researchers to gather additional data points, including information on the dynamic changes of the TG/HDL-C ratio during follow-up. Thirdly, as is customary in observational research, there may be uncontrolled or unmeasured confounding factors that persist, such as the use of antihypertensive, lipid-lowering, uric acid-lowering, antidiabetic medications, immunosuppression or RAS inhibition, even after accounting for known potential confounders like blood pressure and FPG. Nonetheless, we have calculated the E-value to evaluate the potential impact of these unmeasured confounders, and the results suggest that they are unlikely to explain the outcomes. Furthermore, in future research, it would be beneficial to expand the range of variables collected, including antihypertensive, lipid-lowering, SUA-lowering, antidiabetic agents, immunosuppression or RAS inhibition among others. Fourthly, our center started testing blood PLA2R in 2018, while the study population we included started from 2008, so 10 years of PLA2R results are missing. As PLA2R antibody results were missing for 228 of the 363 patients, comprising over 60% of the intended study sample, we didn’t analysis the associations between anti-PLA2R antibody with TG/HDL-C ratio. We will prioritize consistent PLA2R monitoring moving forward in our longitudinal cohort. Once a more robust dataset is available, we will be well positioned to conduct exploratory analyses on relationships of anti-PLA2R levels with TG/HDL-C ratio and other relevant clinical parameters. Finally, it is crucial to acknowledge that this study employed a cross-sectional design, which limits our ability to establish a definitive causal relationship.

## Conclusion

This study provides evidence supporting the TG/HDL-C ratio as an independent risk factor for TA in patients with PMN. Additionally, it reveals a non-linear correlation between the TG/HDL-C ratio and TA risk. Notably, there is a strong association between the TG/HDL-C ratio and TA risk, particularly when the ratio is below 4.25. These findings suggest that reducing the level of the TG/HDL-C ratio could be a sensible approach to mitigating TA risk, regardless of kidney function status. Therefore, it is crucial to prioritize TG management in PMN patients to effectively minimize the risk of TA. Further studies should also explore the mechanisms by which TG/HDL-C ratio influences TA risk and whether interventions aimed at altering this ratio can lead to a tangible reduction in the incidence of TA among PMN patients.

## Data availability statement

The original contributions presented in the study are included in the article/**Supplementary Material**. Further inquiries can be directed to the corresponding authors.

## Ethics statement

The studies involving humans were approved by Medical Ethics Committee of Shenzhen Second People’s Hospital. The studies were conducted in accordance with the local legislation and institutional requirements. Written informed consent for participation in this study was provided by the participants’ legal guardians/next of kin.

## Author contributions

MG: Writing – original draft, Methodology, Resources. LW: Methodology, Writing – review & editing. YC: Investigation, Writing – review & editing. DQ: Investigation, Writing – review & editing. JC: Investigation, Writing – review & editing. HS: Writing – review & editing, Methodology, Formal analysis. HH: Writing – review & editing, Investigation, Methodology, Software. QW: Writing – review & editing, Funding acquisition, Project administration, Supervision.
