# Supplementary material for: Defining the threshold: triglyceride to high-density lipoprotein cholesterol (TG/HDL-C) ratio’s non-linear impact on tubular atrophy in primary membranous nephropathy
Source: Front Endocrinol (Lausanne). 2024 Jan 24;15:1322646. doi: 10.3389/fendo.2024.1322646 (PMC10847559; doi:10.3389/fendo.2024.1322646)
Supplement: Supplementary file 1 [file DataSheet_1.docx]

**Triglyceride to high-density lipoprotein cholesterol (TG/HDL-C) ratio has a nonlinear association with tubular atrophy in primary membranous nephropathy patients**

**Running title: The relationship between TG/HDL-C ratio and TA**

**Mijie Guan^#1,2^, Liling Wu ^1,2^, Yuan Cheng ^1,2^, Dongli Qi ^1,2^, Jia, Chen ^1,2^, Haiying Song ^1,2^, Haofei Hu^* 1,2^, Qijun Wan^* 1,2^**

^1^Department of Nephrology, Shenzhen Second People’s Hospital, Shenzhen 518000, Guangdong Province, China

^2^Department of Nephrology, The First Affiliated Hospital of Shenzhen University, Shenzhen 518000, Guangdong Province, China

*Corresponding author

Haofei HU

Department of Nephrology,

Shenzhen Second People’s Hospital,

No.3002 Sungang Road, Futian District,

Shenzhen 518000,

Guangdong Province,

China

Tel: +86-755-83366388

E-mail: huhaofei0319@126.com

Qijun Wan

Department of Nephrology,

Shenzhen Second People’s Hospital,

No.3002 Sungang Road, Futian District,

Shenzhen 518000,

Guangdong Province,

China

Tel: +86-755-83366388

E-mail: yiyuan2224@sina.com

**Table S1 The baseline characteristics of patients**

| TA | Q1 0.69(0.16-1.06) | Q2 1.37(1.07-1.89) | Q3 2.86(1.90-5.76) | *P*-value |
| --- | --- | --- | --- | --- |
| N | **121** | **121** | **121** |  |
| GENDER |  |  |  | <0.001 |
| Male | 50 (41.32%) | 82 (67.77%) | 85 (70.25%) |  |
| Female | 71 (58.68%) | 39 (32.23%) | 36 (29.75%) |  |
| BMI(kg/m2) | 23.01 ± 3.60 | 24.70 ± 3.92 | 25.77 ± 4.07 | <0.001 |
| DBP(mmHg) | 78.93 ± 11.93 | 83.65 ± 12.69 | 84.86 ± 12.37 | <0.001 |
| HDL.C | 1.93 ± 0.98 | 1.38 ± 0.32 | 1.14 ± 0.27 | <0.001 |
| TG(mmol/L) | 1.26 ± 0.54 | 1.96 ± 0.50 | 3.54 ± 1.31 | <0.001 |
| TG/HDL-c ratio | 0.69 ± 0.24 | 1.43 ± 0.25 | 3.14 ± 1.02 | <0.001 |
| UA(umol/L) | 352.83 ± 87.47 | 382.78 ± 78.73 | 429.88 ± 111.48 | <0.001 |
| UPRO(mg/24h) | 3064.40 (1842.75-5227.00) | 3897.00 (2110.50-6993.14) | 4202.59 (2173.00-8056.22) | 0.044 |
| Hypertension |  |  |  | 0.003 |
| NO | 77 (63.64%) | 54 (44.63%) | 54 (44.63%) |  |
| YES | 44 (36.36%) | 67 (55.37%) | 67 (55.37%) |  |
|  |  |  |  |  |

BMI, Body mass index; DBP, Diastolic blood pressure; TG, Triglyceride; HDL-C, High-density lipoprotein cholesterol; TG/HDL-c ratio; Triglyceride to High-Density Lipoprotein Cholesterol ratio; UA, uric acid; UPRO, 24 h urine protein

**Table S2 Record of obtaining clinical data**

| Clinical | Parameters | Pathologic |  |
| --- | --- | --- | --- |
| ALB(g/L)  TC(mmol/L)  HDL.C(mmol/L)  TG(mmol/L)  LDL.c(mmol/L)  TG/HDL-c ratio  UA(umol/L)  UPRO(mg/24h)  FPG(mmol/L)  eGFR(mL/min/1.73 m2)  HB(g/L) | **GENDER**  **AGE(years)**  **BMI(kg/m2)**  **DBP(mmHg)**  **SBP(mmHg)**  **Smoke history**  **Alcohol history**  **Diabetes history**  **Hypertension** | **Tubular atrophy (%)**  **Global sclerosis (%)**  **Segmental sclerosis (%)**  **Crescent (%)**  **IgG1 (negative-4+)**  **IgG2 (negative-4+)**  **IgG3 (negative-4+)**  **IgG4 (negative-4+)** |  |

**Table S3 Analysis of factors affecting renal tubular atrophy**

| Variable | OR (95%CI)  *P*-value |
| --- | --- |
|  |  |
| Gender |  |
| Male | Ref. |
| Female | 0.78 (0.39, 1.59) 0.4992 |
| Age, years | 1.01 (0.98, 1.03) 0.6715 |
| BMI(kg/m^2^) | 1.01 (0.94, 1.09) 0.7883 |
| Smoke history |  |
| NO | Ref. |
| YES | 1.15 (0.53, 2.50) 0.7173 |
| Alcohol history |  |
| NO | Ref. |
| YES | 0.60 (0.23, 1.51) 0.2742 |
| Diabetes history |  |
| NO | Ref. |
| YES | 0.93 (0.36, 2.43) 0.8829 |
| Hypertension |  |
| NO | Ref. |
| YES | 1.97 (1.06, 3.66) 0.0318 |
| ALB(g/L) | 1.01 (0.96, 1.05) 0.7915 |
| eGFR(mL/min/1.73 m2) | 0.98 (0.97, 0.99) 0.0037 |
| UPRO(mg/24h) | 1.00 (1.00, 1.00) 0.5557 |
| HB, g/L | 0.99 (0.98, 1.01) 0.4702 |
| FPG(mmol/L) | 1.16 (0.85, 1.59) 0.3501 |
| TG/HDL-c ratio | 1.29 (1.04, 1.61) 0.0213 |

BMI, Body mass index; ALB, albumin; eGFR, evaluated glomerular filtration rate; UPRO, 24 h urine protein; HB, hemoglobin; FPG, Fasting plasma glucose; TG/HDL-c ratio; Triglyceride to High-Density Lipoprotein Cholesterol ratio
